# Domoic Acid Epileptic Disease

**DOI:** 10.3390/md12031185

**Published:** 2014-03-06

**Authors:** John S. Ramsdell, Frances M. Gulland

**Affiliations:** 1 Marine Biotoxins Program, Center for Coastal Environmental Health and Biomolecular Research, NOAA, National Ocean Service, Charleston, SC 29414, USA; E-Mail: john.ramsdell@noaa.gov; 2 The Marine Mammal Center, 2000 Bunker Road, Marin Headlands, Sausalito, CA 94965, USA; E-Mail: gullandf@tmmc.org

**Keywords:** domoic acid, amnesic shellfish poison, seizure, epilepsy, aggression, olfactory, piriform cortex, rat, sea lion

## Abstract

Domoic acid epileptic disease is characterized by spontaneous recurrent seizures weeks to months after domoic acid exposure. The potential for this disease was first recognized in a human case study of temporal lobe epilepsy after the 1987 amnesic shellfish-poisoning event in Quebec, and was characterized as a chronic epileptic syndrome in California sea lions through investigation of a series of domoic acid poisoning cases between 1998 and 2006. The sea lion study provided a breadth of insight into clinical presentations, unusual behaviors, brain pathology, and epidemiology. A rat model that replicates key observations of the chronic epileptic syndrome in sea lions has been applied to identify the progression of the epileptic disease state, its relationship to behavioral manifestations, and to define the neural systems involved in these behavioral disorders. Here, we present the concept of domoic acid epileptic disease as a delayed manifestation of domoic acid poisoning and review the state of knowledge for this disease state in affected humans and sea lions. We discuss causative mechanisms and neural underpinnings of disease maturation revealed by the rat model to present the concept for olfactory origin of an epileptic disease; triggered in dendodendritic synapases of the olfactory bulb and maturing in the olfactory cortex. We conclude with updated information on populations at risk, medical diagnosis, treatment, and prognosis.

## 1. Case Definition

Domoic acid epileptic disease is characterized by spontaneous recurrent seizures weeks to months after domoic acid poisoning and unusual behaviors in animal subjects, notably conspecific aggression.

## 2. Concept of Domoic Acid Epileptic Disease

Domoic acid poisoning is well characterized in humans (amnesic shellfish poisoning, see [1]) and the California sea lion (domoic acid toxicosis, see [2]) and investigated in experimental animals as detailed in several reviews [3,4,5,6,7]. It is also evident that domoic acid can trigger a separate process of epileptogeneis, which, over a latent “silent” period of weeks to months, causes a disease state of progressive recurrent seizures and behavioral abnormalities [8,9,10,11]. We now define domoic acid epileptic disease as distinct consequence of domoic acid poisoning that occurs in the absence of domoic acid. The term “disorder” is commonly associated with the many forms of idiopathic and symptomatic epilepsy, in part because the malfunctioning is the result of a progressive change in neuronal reorganization known as epileptic maturation [12,13]. However, in the case of domoic acid, we use the term “disease” because it is caused by a known external factor with a defined sequence of events and course of prognosis. This concept report focuses on those essential characteristics of domoic acid poisoning that lead to epileptic disease drawing on case studies in humans and sea lions and a rat model to elucidate the structural basis specific to domoic acid epileptic maturation.

## 3. Acute Poisoning

Acute poisoning in humans has been characterized as amnesic shellfish poisoning [1] and in marine animals as domoic acid toxicosis [2] and is a requisite step that initiates the epileptogenic process to progress to an observable disease state. Acute poisoning is discussed briefly here to provide context to understand the clinical presentation and neurobiology of domoic acid epileptic disease.

### 3.1. Amnesic Shellfish Poisoning

Domoic acid poisoning was first reported as amnesic shellfish poisoning (ASP) after an outbreak of illness from 11 November to 4 December 1987, focused in Quebec Province [14]. The case definition was any individual who consumed mussels harvested from Prince Edwards Island River estuary after 1 November 1987, developed either gastrointestinal symptoms within 24 h, *i.e.*, vomiting, diarrhea, and/or abdominal cramps, or at least one neurological symptom within 48 h, e.g., confusion, memory loss, disorientation, or other major objective sign, such as seizures, coma, or cranial nerve palsies. The ASP event included 107 documented and 38 probable cases. Eighteen percent of cases were hospitalized and half of these were in intensive care, with four deaths within 24 days. Seizures were found in the most severely poisoned individuals and became progressively less frequent over an eight-week period. Neurological batteries of tests on 14 of the most severe patients four months after poisoning identified a selective and severe impairment of anterograde memory in twelve individuals, peripheral neuropathy in eleven, and altered metabolic activity in the temporal lobes of four individuals. [15]. Three case studies describing different clinical features have been reported [16].

### 3.2. Acute Domoic Acid Toxicosis

Undefined mortality events with signs of neurological poisoning of California sea lions (*Zalophus californianus*) have been reported over multiple years, with domoic acid identified as a causative agent in 1998. That year, 400 sea lions were found stranded onshore from Monterey Bay to San Diego and at least 70 animals were examined [17]. The poisoning was correlated with a late spring bloom of the diatom *Pseudo-nitzschia australis*, domoic acid contaminated anchovies, and domoic acid in the urine of affected sea lions and other marine species [18]. Clinical signs in sea lions included ataxia, head weaving, seizures, or coma. Seizures varied in severity but were continuous during the period of toxicosis, lasting about one week, followed by treatment-aided recovery or death. Detailed clinical evaluations for acute domoic acid toxicosis in sea lions have been reported [2].

## 4. Domoic Acid Epileptic Disease

### 4.1. Human Case Study

Domoic acid epileptic disease was first recognized in a case study of temporal lobe epilepsy after the 1987 amnesic shellfish poisoning event in Quebec. Nearly a year after the ASP event, an 84 years old male survivor re-experienced severe seizures and was diagnosed with temporal lobe epilepsy caused by domoic acid intoxication [8].

#### 4.1.1. Clinical Evaluation

This individual initially experienced disorientation after eating the contaminated mussels, followed by nausea, vomiting, confusion and coma. Three days after poisoning, focal seizures progressed to complex partial status epilepticus. These seizures were nonresponsive to the mild anticonvulsant phenytoin, yet, responded to large doses of phenobarbitol and resolved by three weeks. Periodic epileptiform discharges continued with dissipating fronto-temporal slow wave electrogenic abnormalities. No structural abnormalities were evident by brain computed tomographic (CT) scan. He was discharged from the hospital seizure-free four and half months after the poisoning event with severe anterograde memory deficit. The subject continued with phenobarbitol management for two additional months and had a normal EEG at eight months. One year after the poisoning event, the man exhibited an acute episode of focal seizures with clonic contraction of the left arm and leg. These seizures responded to phenytoin and were controlled with continuing pharmacotherapy. One and a half years later he developed pneumonia and died.

#### 4.1.2. Neuropathological Findings

Neurohistochemistry was conducted on five of the Montreal ASP event patients; four patients dying at intervals of 7, 12, 24, and 98 days after poisoning and the one patient referenced above who died three years after the event [19]. The neuropathology of the four patients was similar showing severe damage to the hippocampus throughout the pyramidal cell layer other than H2 (*i.e.*, CA2) and in the dentate granule cell layer of three subjects and into the subiculum. All cases also had damage to the amygdala, piriform cortex and thalamus with some damage to the septum, olfactory tubercle, and claustrum and nucleus accumbens. Neuronal damage progressed from eosinophilic neurons and fine vacuolation at seven days, phagocytosis of neurons at 12 days to disappearance of damaged neurons and collapse of the pyramidal cell layer at 24 and 98 days. A MRI of the fifth subject, the single case study for epileptic disease, upon his recurrence of seizures one year after poisoning identified bilateral hippocampal atrophy, and PET imaging identified a reduced bi-temporal glucose metabolic rate [8]. Two years later, postmortem gross examination revealed severe bilateral hippocampal sclerosis [8]. Histochemistry found extensive damage similar to that seen in the subjects shortly after the poisoning event, with total to extensive loss of pyramidal cell layers, with some sparing of H2 and additional necrosis in the amygdala, septum, secondary olfactory areas, and nucleus accumbens.

#### 4.1.3. Kainic Acid Model for Temporal Lobe Epilepsy

This single case study provided observational evidence that domoic acid induced status epilepticus can progress after a latent period to temporal lobe epilepsy (TLE) due to mesial temporal sclerosis. However, it was of interest to epilepsy research in that it provided a real case scenario for an important experimental model for human TLE. The kainic acid model of experimental epilepsy has yielded a detailed understanding of some of the processes involved in human acquired epilepsy. Acquired, or “injury-induced”, epilepsy accounts for about half of human TLE and can be caused by status epilepticus, febrile seizures, stroke, or head injury [20]. When administered systemically in rats, kainic acid induces status epilepticus and produces an epilepsy syndrome similar to human TLE. [21] This model has three stages: (1) an initial toxic insult that manifests as a several hour episode of status epilepticus; (2) a period of “silent toxicity” normally spanning several weeks with no outward seizure activity; and (3) the development of a permanent state of spontaneous recurrent seizures, the hallmark of the epileptic state [22]. Although kainic acid acts via specific endogenous kainic acid receptors to produce the neurological sequelae [23], this model has been critiqued because the inducing agent is a neurotoxin. The single domoic acid human case study therefore provided an unexpected “real life” context in which to view the kainic acid model [8].

### 4.2. California Sea Lion Chronic Epileptic Syndrome

Domoic acid epileptic disease was discovered as a chronic epileptic syndrome in California sea lions over the course of examining domoic acid toxicoses cases from 1998 to 2006 [9]. The sea lion study provided a breadth of insight into clinical presentations, unusual behaviors, brain pathology and epidemiology. Of the 715 sea lions that stranded with neurological symptoms over this period, nearly one quarter of the animals did not fit the criteria for acute poisoning events. These animals instead stranded individually rather than en masse at times when no domoic acid producing algal blooms were detected in the area, expressed intermittent seizures and unusual behaviors, with strandings of individuals peaking approximately four months after an acute poisoning event. These animals were designated as chronic neurological cases.

#### 4.2.1. Clinical Evaluation

One hundred-twelve of the chronic neurological cases were observed to develop intermittent seizures varying in duration from hours to weeks. Another 32 animals treated for acute poisoning developed intermittent seizures after a seizure free period ranging from two to twelve weeks. Additionally, six animals initially admitted for acute poisoning and released were readmitted with intermittent seizures and undetectable levels of domoic acid. The seizures observed were generalized from simple or focal seizures to tonic-clonic seizures, with the clonic phase commonly showing extension of flippers and the neck and often resulting in convulsive status epilepticus. Seizures were often preceded by vomiting, pacing, or swimming in tight circles. Animals also presented with periods of marked lethargy and inappetence, vomiting, muscular twitching, and central blindness. Abnormal behaviors included stranding in atypical locations, stereotypic behaviors such as scratching and aggression towards conspecifics or humans that was atypical of animals in rehabilitation [9].

#### 4.2.2. Neuropathological Findings

Eighty-four sea lions, nearly all adult females that died between one day and ten months after admission for domoic acid toxicosis, were examined by histochemistry [24]. A peracute finding only in animals examined within one day of admission was laminar vacuolization of the thin lacunosum and moleculare strata in area CA3. This layer receives two major excitatory pathways; dendrites of pyramidal cell receiving input fibers from the perforant path of the entorhinal cortex and mossy fibers of the dentate gyrus granule cells. Neuronal necrosis was prominent in this group, as well as in animals surviving up to one week. Necrosis was visible in the hippocampus (most notably dentate granule cells and CA3 sector pyramidal cells), the amygdala, and additionally in some animals the olfactory bulb, pyriform lobe, and rostral thalamic nuclei. As time progressed, histochemical analysis of animals surviving between one week and ten months, showed a smaller extent of active necrosis; however this damage remained evident as hippocampal atrophy.

Histochemical analysis of 87 animals meeting the definition of chronic neurological disease found frequent atrophy in the hippocampal formation and a few instances of active neuronal necrosis [9]. The atrophy occurred first in the Ammon’s horn followed by the parahippocampal gryus, likely reflecting cell loss in the mid layers of the entorhinal cortex that project perforant path fibers to the dentate gyrus and CA3 region. Atrophy of the hippocampi and parahippocampal gyri was most commonly asymmetrical and abnormal hippocampal formation was also described in 41 out of 42 animals tested by MRI. Specifically, mild to severe hippocampal atrophy was common and often associated with thinning of the parahippocampal gyrus, enlargement of the inferior horn of the lateral ventricle and abnormal intense T2 signals in the parahippocampus. Sea lions with acute toxicosis and chronic disease were also analyzed by MRI, showing the same features of hippocampal atrophy and parahippocamal thinning that increased during the progression of disease.

Quantitative analysis of the hippocampus of sea lions with epileptic disease revealed cell loss in the granular cell layer, hilus, and pyramidal cell subfields similar to patients with temporal lobe epilepsy [25]. At the gross level, unilateral hippocampal sclerosis was common both the sea lion and human subjects. A higher resolution of inspection identified evidence for synaptic reorganization in the sea lions as commonly found in human temporal lobe epilepsy, including loss of somatostatin-immunoreactive hilar cells with appearance of somatostatin-immunoreactive axons in the dentate gyrus, as well as mossy fiber sprouting. A separate immunohistochemical analysis of sea lion hippocampus has shown evidence of oxidative stress in pyramidal cells of CA1 and subiculum and dentate granule cells animals with acute toxicosis and chronic neurological disease and a decrease in glutamine synthetase in animals with chronic neurological disease [26].

The histopathology of domoic acid epileptic disease is less apparent in younger animals [9,27] and this is discussed further under the section Populations at Risk.

### 4.3. Rat Model for Domoic Acid Epileptic Disease

**Figure 1:**
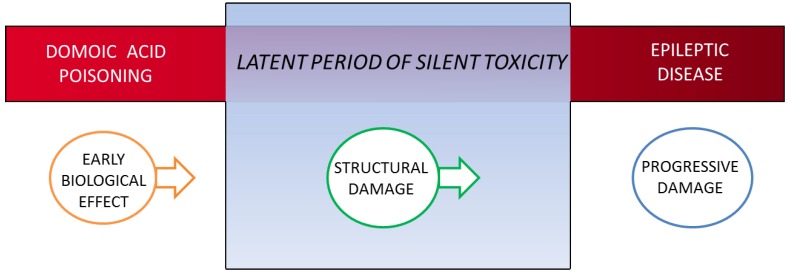
Three stage progression of domoic acid poisoning to epileptic disease. A latent period of silent toxicity characterizes the transition between domoic acid poisoning and epileptic disease. The continuum from exposure to disease can be described stepwise from early biological effect seen during the poisoning event to altered structure/function during the latent period to progressive damage that intensifies with the appearance of clinical disease.

The application of an experimental rodent model for domoic acid epileptic disease has proved useful to better understand the maturation of the disease state and its relationship to its unusual behavioral manifestations. The sequelae for domoic acid epileptic disease in the rat model begin with (1) an early biological effect precipitated by domoic acid poisoning; its resultant (2) structural damage that alters function during the latent phase and; and (3) progressive damage that expresses manifestations of the disease state (Figure 1). This sequence of events parallels the progression of temporal lobe epilepsy proposed by [12] of (1) epileptogenic lesion; (2) reorganization during epileptogenesis and; (3) reorganization continuing owing to recurrent seizures. The rat model has identified a structural basis for the sequela for epileptic disease under the minimal exposure of domoic acid to induce a progressive epileptic state.

#### 4.3.1. Early Biological Effect

The emergence of domoic acid disease in a human subject, an epilepsy-like syndrome in sea lions and the wide utilization of a rat model using kainic acid as a tool for epilepsy research [8,9,28] all beckoned for an experimental model for domoic acid epileptic disease. However, domoic acid was not initially suitable for an epilepsy model due to its high lethality. A single experimental exposure to domoic acid at levels sufficient to induce status epilepticus was lethal in marmosets, even in the presence of agents to control seizures and at doses below the level needed to cause status epilepticus failed to lead to epileptic behavior [29]. To combat domoic acid lethality, a modified triturated dosing protocol, similar to an established kindling protocol for kainic acid in rats, [30] was found suitable to induce a nonlethal status epilepticus with domoic acid [10]. Using this protocol 94% of rats developed a spontaneous and recurrent seizures within six months, indicating that a single episode of status epilepticus is the early biological effect for domoic acid epileptic disease.

Domoic acid induced status epilepticus kindling is obtainable with an initial sub-lethal dose of 1.0 mg/kg intraperitoneal injection, which characteristically leads to stereotypic scratching within 30 min. [10]. A second 1.0 mg/kg dose is given an hour later. Doses after that time are tailored to achieve a minimal number of doses to lead to status epilepticus and assure survival. A half dose was given if the rat showed early signs of seizures such as foaming, staring, or atypical posturing. Hourly dosing was stopped when an animal exhibited its first motor seizure as determined by forelimb clonus, a stage III convulsion adapted from the modified Racine scale [30], within the hour following the last dose (Figure S1, Supplementary Information). Status epilepticus is determined to be the point at which the animals exhibit one or more stage IV (forelimb clonus with rearing) or V (forelimb clonus with rearing and loss of balance) seizures per hour for three consecutive hours [10]. Rats recover from domoic acid induced status epilepticus and enter an extended latency period without observable symptoms before the subsequent appearance of spontaneous recurrent seizures.

#### 4.3.2. Structural/Functional Damage Evident in the Latent Period

**Figure 2:**
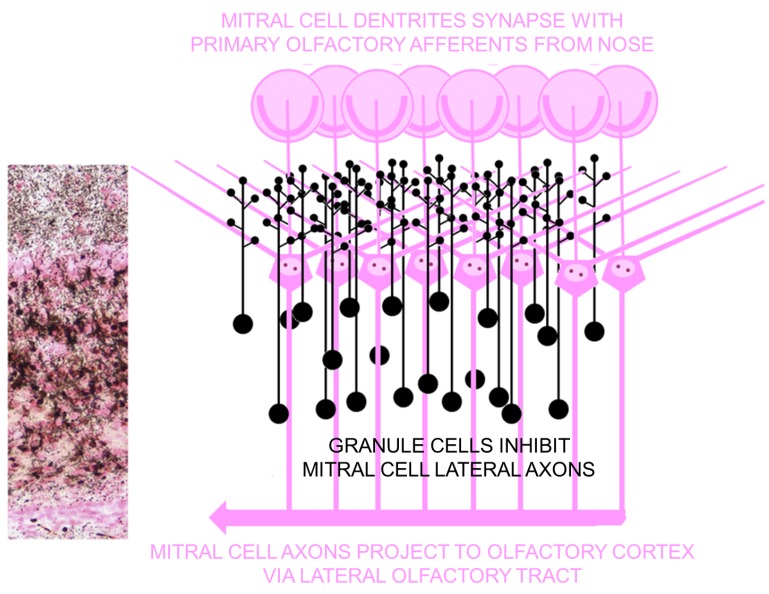
Widespread domoic acid damage in olfactory bulb is localized to granule cells. Left: Photomicrograph of coronal section of olfactory bulb stained by amino cupric silver histochemisty with neutral red counterstain. Black amino cupric silver impregnates soma and dendrites of granule cells and neutral red stains soma, axons, and terminals of mitral cells. Additional magnifications of the field this photomicrograph and labeled subdivisions can be found in Figure S2, Supplementary Information. Right: schematic diagram corresponding to photomicrograph showing silver impregnated (black) granule cells and dendritic contacts on lateral dendrites of mitral cells. Schematic after [32].

Structural damage resulting from domoic acid induced status epilepticus that can alter function during the latent period has been characterized using degeneration specific cupric-silver histochemistry [31]. The most extensive damage was found in the olfactory bulb, specifically granule cells (Figure 2) and related olfactory pathways, including the anterior/medial olfactory cortices, endopiriform nucleus and entorhinal cortex (Figures S2–S6, Supplementary Information). Although the hippocampus is the most prominently recognized target of domoic acid damage in the brain, when using the protocol described above, the damage to the olfactory pathways is more extensive than that found in the hippocampus.

**Figure 3:**
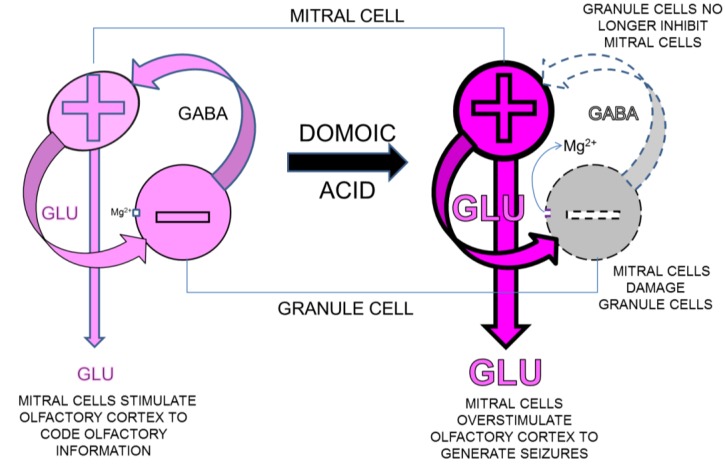
Proposed mechanism for domoic acid induced damage to granule cell dendrodentric contacts with mitral cells in olfactory bulb in rat model for domoic acid epileptic disease. Left: Activation of mitral cells releases glutamate (GLU) into the dendrodentric space. The depolarization of dendritic spine of granule cells relieves magnesium block on NMDA receptor allowing glutamate induced calcium entry and release of GABA. GABA feeds back to hyperpolarize lateral dendrites of mitral cells. Right: Excitotoxic damage of dendritic spines (broken lines) disrupts GABAergic feedback at dendrodentric contacts to promote hyperactivity (bold lines) of mitral cells lack due to loss of inhibition of lateral dendrites.

We propose that domoic acid induced damage to the dendritic spines of olfactory granule cells is the proximal site of domoic acid action and as such triggers excitability of mitral cells and activates downstream pathways of the olfactory cortex (Figure 3). Excitotoxic damage these olfactory pathway damage can be sufficient to initiate epileptogenesis, an activity-dependent reorganization occurring during the latent phase in the absence of domoic acid. This activity-dependent reorganization eventually expresses a chronic state of progressive epileptic disease, similar to that observed in the chronic neurological disease of the California sea lion.

#### 4.3.3. Behavioral Changes during Expression of Disease State

Several weeks after the status epilepticus, two main manifestations are seen: rats develop a progressive state of seizures similar to epilepsy or atypical aggressive behaviors, with the majority of rats showing various degrees of both responses [11]. Spontaneous recurrent seizures occurred in 94% of rats within six months with an average latent period of five weeks [10]. Retrospective analysis of archived video collected for 12 weeks after status epilepticus revealed the second disease manifestation state of increased aggression [11]. Serious fighting behaviors were observed weeks after status epilepticus, including biting and wrestling, in excess of what is normal for establishing dominant/subordinate relationships and not generally not seen among established colonies or cage mates [33]. This aggression between cage mates, more typically seen when introduced to an intruder rat [34], appeared after a period of latency (one to three weeks), reached a predominance of the more aggressive behaviors (weeks six to eight) that transitioned to less aggressive behaviors in weeks 9, followed by a general decline in overall aggression. Seizure activity and aggression peaked six weeks after status epilepticus; however, there is a lack of correlation between each disease manifestation and some animals exhibit only one or the other. This insight into seizures and behavior provided the first experimental evidence that the unusual aggressive behavior observed in California sea lions can also originate from domoic acid poisoning and is not necessarily a consequence of observable convulsive seizures.

#### 4.3.4. Structural Damage during Expression of Disease State

Progressive structural damage to the brain is more limited than that found after domoic acid induced status epilepticus [35]. Cupric silver staining, reflective of recent (within several days) neuronal necrosis, was examined to identify ongoing damage in animals that progressed to spontaneous recurrent seizures and/or atypical aggressive behaviors. Positive silver staining is most evident in the deep layers of the piriform cortex, including large cells and terminal fields in layers 2 and 3 and terminal fields in the posteromedial cortical amygdaloid nucleus (Figure S7, Supplementary Information). A pronounced thinning in these cellular layers due to cumulative neuronal loss is evident by additional Nissl staining (Figure S8, Supplementary Information). The piriform cortex is highly seizure prone and a likely origin for the development of widespread limbic seizures [36,37]. Damage and cell loss in this region indicate continued cellular damage after the status epilepticus insult and is consistent with the hypothesis that both the epileptic and aggressive states may originate from seizure activity as reflected in the progressive damage to deep layers of the piriform cortex [38].

Although the piriform cortex is a common site of progressive damage in both spontaneous recurrent seizures and atypical aggression, damage to cell bodies, and terminal fields of the anterior olfactory cortex was selective for aggressive rats [31]. This region has been proposed to modulate conspecific social recognition by olfactory modality [39] and the ability to distinguish conspecifics (*i.e.*, colony members) from intruders preempts overt aggressive behaviors [33]. If continued damage to the anterior olfactory cortex altered this population of cells, animals would have a deficit in the ability of short-term conspecific recognition and this deficit could promote the aggressive behavior seen in domoic acid epileptic disease.

### 4.4. Concept for Olfactory Origin of Epileptic Disease

#### 4.4.1. Parallels between Olfactory Cortex and Hippocampal Formation

**Figure 4:**
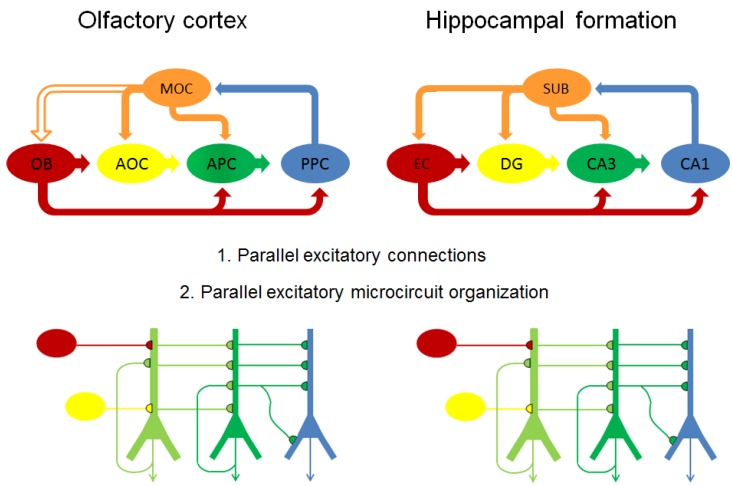
Parallels between olfactory and hippocampal pathways. Top: schematic flow diagrams of excitatory (solid arrows) connections within olfactory cortex (left) and hippocampal formation (right). Connection between MOC and OB is inhibitory and shown as open arrow. Bottom: schematic diagram of excitatory microcircuit organization of olfactory cortex (left) and hippocampal formation (right). Color coding show corresponding anatomic regions in olfactory cortex and hippocampal formation and color coding is consistent for anatomic regions in top and bottom figures. OB: olfactory bulb; AOC: anterior olfactory cortex; MOC: medial olfactory cortex; APC, anterior piriform cortex; PPC, posterior piriform cortex; EC: entorhinal cortex; DG, dentate gyrus; CA3: Cornu Ammonis-3 subfield; CA1: Cornu Ammonis-1 subfield; SUB: subiculum. After [42].

Structural damage to the hippocampal formation is consistently reported after domoic acid exposure and is the subject of numerous focused investigations that have been reviewed [3,5]. However, structural damage is commonly widespread, notably in the amygdala, septum, midline thalamic nuclei, and olfactory bulb and its cortical fields [24,40,41]. The olfactory cortex and hippocampus formation share many parallels. These three layered cortices expanded in early rodents to enhance processing of olfactory and tactile spatial patterns needed for a nocturnal environment [42]. Reassessment of the structure and interconnections within olfactory pathways has given rise to recognition of a dominant forward flow pathway along the lateral olfactory tract from the olfactory bulb to piriform cortex with recurrent self excititatory feedback and back projections [43]. This parallel-distributed network of intrinsic and extrinsic excitatory connections and microcircuit organization has close structural parallels with the hippocampal formation (Figure 4) [43,44].

#### 4.4.2. Seizures Originating in Olfactory Bulb

Repeated stimulation of brain loci associated with the limbic system including the hippocampal formation, septum, amygdala, and the olfactory bulb develop (kindle) higher grade seizures resulting in atypical and convulsive behaviors [45]. These are the same loci commonly damaged after domoic acid exposure [40,41]. The olfactory bulb although not commonly associated with epilepsy has a unique role as an initiator of seizures. It initiates odor induced seizures [46] as well as propagates kindling in response to direct application of kainic acid [47]. The olfactory bulb is among the most sensitive brain regions to systemic administration of domoic acid [48] and hence it is not surprising that it is a primary target to initiate status epilepticus in the rat [31]. The piriform cortex, on the other hand, contains the most sensitive circuits to electrical and chemical induction of seizures and has been proposed to play a central role to intensify the spread of seizures [36,37]. This is consistent with the finding that piriform cortex, which receives direct input from the olfactory bulb via the lateral olfactory tract shows continued damage during maturation of domoic acid epileptic disease [35].

#### 4.4.3. Olfactory Outcome Pathway for Rat Model for Domoic Acid Epileptic Disease

**Figure 5:**
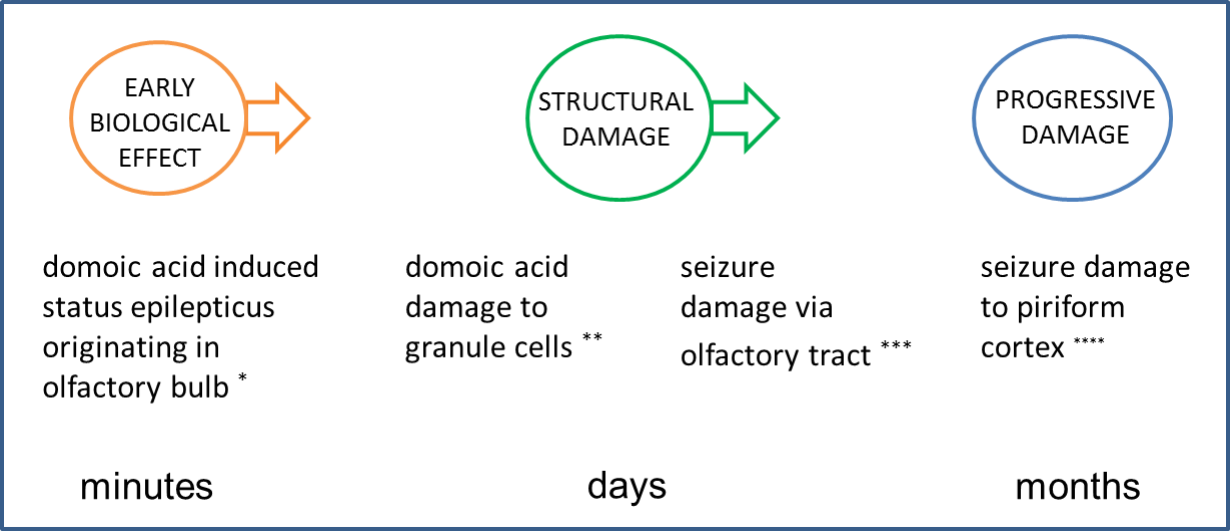
Proposed outcome pathway based upon rat model for olfactory involvement domoic acid epileptic disease. Sequences are illustrated by photomicrographs of behaviors or structural damage in accompanying Supplementary Figures (* Figure S1; ** Figure S2; *** Figures S3–S6; **** Figures S7 and S8).

An outcome pathway for domoic acid induced epileptic disease points to prominent, but certainly not exclusive, involvement of olfactory circuits in the rat (Figure 5). In the rat, the granule cells of the olfactory bulb are a sensitive target for low doses of domoic acid required for status epilepticus without acute lethality. A reasonable initiation for epileptogeneis is selective damage to these cells leading to uncoupling of GABAergic inhibition of mitral cells in the outer plexiform layer of the olfactory bulb. This would initiate a sequela of increasing excitation of mitral cells transmitted in large part via the lateral olfactory tract to olfactory cortical areas. Excitation-dependent reorganization can facilitate progressive recurrent spontaneous seizures and correspond to the continuing damage as seen in deep layers of the piriform cortex and terminal fields of the posteromedial cortical amygdaloid nucleus.

#### 4.4.4. Role for Hippocampus and Olfactory Bulb in Domoic Acid Epileptic Disease

Structural damage to hippocampal formation is clearly a consequence of domoic acid toxicity and correlative to acute seizures and memory impairment. Cellular loss especially in pyramidal cells of Ammon’s horn that over time leads to atrophy, collapse and sclerosis are common pathological markers for sea lion and human subjects with temporal lobe epilepsy. Examination of the hippocampus also reveals synaptic reorganization in hilar and dentate granule cells indicative of synaptic reorganization and formation of recurrent excitatory circuits [25]. Collectively, these findings point to the hippocampal formation and notably the resprouting of mossy fibers, as a locus for epileptogeneis and maturation of domoic acid epileptic disease.

In so much that domoic acid poisoning provides a real world view for a defined cause of epilepsy, an experimental model showing the same disease progression and atypical behavioral outcomes, provides a controlled examination for epileptogeneis and maturation of this disease state [28]. The dominate role of olfactory pathways in this experimental model may reflect a protocol or a species bias. For example, the sensitivity of olfactory *vs.* hippocampal targets for domoic acid may be more prominent in rodents than in sea lions. However, sea lions show comparable atypical aggressive behavior during domoic epileptic disease as seen in rats, which appears to reflect a deficit in olfactory processing of conspecific recognition [35,49]. Thus closer examination of the olfactory pathways is merited in cases of domoic acid epileptic disease.

## 5. Populations at Risk

Domoic acid toxicity has more pronounced effects on aged individuals as evidenced in both human and California sea lion cases. A preponderance of affected individuals in the 1987 human poisoning event were older than 40. In the California sea lion, acute domoic acid toxicosis is found largely in adult females, whereas the resultant epileptic disease is found most commonly in young animals of both sexes. The bases to susceptibility include environmental and age-dependent physiological factors as described below.

### 5.1. Observational Studies

#### 5.1.1. Human Studies

Analysis of 145 ASP cases showed no sex difference, but were skewed by 80% to age 40 and older. Nearly all of the most severely affected, including 11 of 13 treated in intensive care units and four fatalities, were over 68 years old, including the 84-year-old male that progressed to having epilepsy as previously discussed. Two other individuals severely poisoned had compromised kidney function due to chronic renal disease. Collectively, this indicates age and renal function are predisposing factors for ASP in humans.

#### 5.1.2. Sea Lion Studies

Analysis of 551 cases of domoic acid toxicosis in sea lions, between 1998 and 2006, shows a strong sex and age class distribution, with 70% of cases found in adult females. By contrast, analysis of 164 cases of chronic epileptic disease showed only 30% to be adult females. While adult females were 2.3 times more frequently diagnosed with acute poisoning, other age classes were more commonly found with chronic disease, most largely represented by younger sea lions of both sexes.

The preponderance of adult female sea lions acutely poisoned by domoic acid reflects their year round occupancy at rookeries positioned in feeding zones impacted by toxic *Pseudo-nitzschia* blooms. By contrast, males spend only a short period at the rookeries in June to breed, without feeding during the breeding season, before migration out of the region heavily impacted by these blooms. Given a gestation period of nearly a year and mating each summer, female sea lions spend much of their adult life span pregnant or nursing. This contributes to the extensive amount of reproductive failure seen in California sea lions in the last decade that has been partially associated with domoic acid producing *Pseudo-nitzschia* blooms [50]. The 1998 event occurred during the last month of gestation and of the 54 adult females admitted for rehabilitation, nearly one-third experienced prenatal reproductive failure [51]. An even larger event in 2002, which also occurred just prior to term of parturition, resulted in 209 documented cases of domoic acid associated reproductive failure [50]. A subset of these animals had measurable levels of domoic acid in amniotic fluid, indicating that not only are pregnant females poisoned by domoic acid, but their fetuses may be at substantially greater risk.

The symptomatology of epileptic disease differs between adult and younger animals [9]. One third of younger animals did not have seizures at admission and overall displayed lower grade symptoms of tremors, ataxia, or depression. The younger animals that died had minimal or atypical neuropathological damage and lacked the hippocampal sclerosis and limbic system damage observed in adult sea lions. This difference in disease presentation seen in young sea lions thus appears related to the developmental stage at which domoic acid exposure occurs and is discussed in further detail in the section Intrinsic Susceptibility Factors.

### 5.2. Toxicokinetic Susceptibility Factors

#### 5.2.1. Renal Clearance

Renal clearance is a primary predisposition factor for domoic acid toxicity in humans. Clearance studies following intraparental administration in rats indicate that domoic acid remains largely intact and is cleared rapidly by the kidneys with a renal clearance of less than 10 mL/min/kg [52]. By contrast, monkeys have a clearance rate about ten times less than that of rats [53]. The clearance of domoic acid by the kidneys is resistant to probenecid, an inhibitor of reabsorption of organic acids in renal tubules, which indicates that domoic acid is cleared largely by glomerular filtration [52].

#### 5.2.2. Maternal Toxicokinetics

The high occurrence of reproductive toxicity in pregnant sea lions appears accountable in large part by poisoning events within the foraging area of rookeries of adult females; however, toxicokinetic factors may also play a role. Analyses of two independent toxicokinetic studies, one on non-pregnant rats [53] and the other on late term pregnant rats [54], indicate a higher exposure risk during pregnancy. The extended residence time and slower elimination during pregnancy may be due to fetal recirculation of domoic acid.

#### 5.2.3. Prenatal Toxicokinetics

In the pregnant rat, domoic acid in maternal plasma readily crosses the placenta to enter the fetus and is retained in the amniotic fluid [54], with maternal-fetal transfer of 24% between the plasma compartments [55]. Domoic acid was found in the amniotic fluid of sea lions up to eight days after stranding, which suggests that amniotic fluid acts as a sink for domoic acid in sea lions as well [50]. The longer fetal retention of domoic acid, particularly in amniotic fluid, indicates that the fetus may be continually re-exposed during gestation, resulting in a substantially larger total exposure over time. This is reflected in a longer retention of domoic acid in fetal brain indicating high susceptibility of the fetus to domoic acid [55].

#### 5.2.4. Postnatal Toxicokinetics

Maternal plasma domoic acid also readily enters the milk, posing a potential hazard during the lactation period. Analysis of domoic acid transfer during lactation in the rat indicates that milk concentrations reach 6% of maternal plasma levels at one hour [56]. However, the efficiency of oral absorption by the postnate of domoic acid in milk is very low, with an estimate of a less than 0.1% [56]. Hence, domoic acid exposure to the postnate does not pose as a significant level of susceptibility as does *in utero* exposure in the prenate.

### 5.3. Intrinsic Susceptibility Factors

Key to risk in populations at greater exposure, either through environmental exposure or internal disposition, is intrinsic susceptibility. The effects of domoic acid and the related excitotoxin kainic acid have been examined both during aging and during neurodevelopment. Of importance in comparing studies in rodents of clinical disease in humans or sea lions is the appropriate scaling of development and ageing [57,58].

#### 5.3.1. Age Susceptibility

A foundation study to age susceptibility was conducted by Wozniak *et al.* [59] who compared the effects of kainic acid on three age groups of rats: young rats 5–6 months of age, year old rats, and aged two year old rats. These age groups in rats correspond roughly to sexual maturation, adult and aged humans and sea lions. Young rats had little or no response to the doses that caused seizures and neurological damage in the middle aged rats. The aged rats had a huge toxic response and quickly died, due to their reduced renal clearance. The greater sensitivity of aged rats due to decreased toxin elimination has been confirmed for domoic acid [60] and is consistent with the finding for human poisoning and disease. The different presentation of juveniles and yearling sea lions with epileptic disease with lower grade seizures and less structural damage to brain is consistent with the experimental studies with kainic acid; however, the precise roles of toxicokinetic or intrinsic susceptibility factors remain to be determined [9,27].

#### 5.3.2. Developmental Susceptibility

A developmental basis for domoic acid induced epileptic disease has been suggested for humans and sea lions and has been characterized in rodent models [5,57,61,62]. Research data on developmental toxicity of domoic acid in mice and rats indicate three potential types of toxicity related to major neurological milestones.

The first milestone, neurogenesis, occurs in the last third of gestation in rodents and the second quarter of sea lion *in utero* development [57]. Poisoning during neurogenesis is anticipated to lead to a neuronal migration toxicity expressing a disease state with characteristics similar to that described by Dakshinamurti *et al*. [63] or Levin *et al*. [64]. In short, animals would birth asymptomatically for spontaneous recurrent seizures but with an increased excitability of the brain to stimulatory agents, such as exposure to domoic acid later in life and a decreased cognitive reserve.

As neurogenesis and migration reaches completion, the brain begins the second development milestone, synaptogenesis. The synapses are formed and the brain undergoes growth traditionally referred to as the brain growth spurt [65]. This second milestone occurs from birth to the first three to four weeks of postnatal life in rodents and approximately the third quarter of gestation in sea lions [57]. Poisoning during synaptogenesis is expected to lead to animals developing with an additional spectrum of learning deficits and increased emotionality behaviors. Poisoning during synaptogenesis would likely express a disease state with low grade seizure behaviors, more likely the result of novelty induced seizure response to stressful situations [66] than spontaneous recurrent seizures. These animals would likely express a disease state with characteristics similar to those as described by Doucette *et al*. [66,67].

The third milestone corresponding to maturation of dentritic spines and full limbic discharges leads to excitotoxin induced status epilepticus that progresses to epileptic disease. This occurs approximately six weeks of postnatal life in rats [30] and, perhaps, as early as the end of pregnancy in the sea lion [57]. A single status epilepticus is the key step leading to domoic acid induced epileptic disease in rats. The rat model described by Muha and Ramsdell [10] induces status epilepticus in seven week old rats and follows progression of disease out to eight months of age corresponds to exposure shortly after brain maturation and development epileptic disease into adulthood.

## 6. Medical Diagnosis and Treatment

### 6.1. Diagnostic Criteria

Domoic epileptic disease is characterized by spontaneous recurrent seizures, weeks to months after domoic acid poisoning and unusual behaviors in animal subjects, notably conspecific aggression. The diagnostic criteria for domoic acid poisoning and domoic acid toxicosis in sea lions have been well defined. For human ASP the criteria are a person who consumed shellfish confirmed or suspected to contain domoic acid who developed any of a subset of gastrointestinal symptoms within 24 h. or neurological symptoms within 48 h. [1]. For acute domoic acid toxicosis in sea lions, a combination of clinical signs, the presence of domoic acid in fluid samples and epidemiological findings are used for diagnosis [51]. Stranding of sea lions when there are blooms of domoic-acid producing algae *Pseudonitzschia australis* offshore with clinical signs of seizures, ataxia, head weaving, decreased responsiveness to stimuli, and scratching behavior is typical [2].

### 6.2. Diagnostic Tests

Identification of domoic acid in consumed food or body fluids is the most definitive diagnostic test. Domoic acid can be measured by several methods. Rapid screening by enzyme-linked immunosorbent assay and confirmation by tandem liquid chromatography-mass spectrometry are current methods of choice. In the case of the 1987 ASP event, contaminated mussels were found to have between 31 and 128 mg domoic acid/100 g shellfish [1]. Domoic acid was below the detection limit in collected plasma samples, due to both delay in sample collection and the rapid elimination of toxin. In the case of the sea lion epizootic, domoic acid was detected in excess of 200 ng/mL in urine, plasma, gastric fluid and fecal samples, and, in the case of pregnant animals, in amniotic fluid [17,50]. However, samples are not always available before the toxin is cleared in domoic acid toxicosis and domoic acid is no longer present in body fluids when poisoning progresses to the epileptic disease state. Serum chemistry has identified markers consistent with seizure activity including elevated hematocrits, eosinophil counts and creatine kinase [2]. A study of control, domoic acid toxicosis and epileptic disease animals identified eosinophil counts as a diagnostic biomarker for domoic acid exposure [68].

Diagnosis of domoic acid toxicosis and epileptic disease is frequently supported by neuropathological changes. Post-mortem histology of adult subjects provides detailed identification of damage to specific brain regions and, in the sea lion, has been graded by time after exposure [24]. Neuropathogical changes, electromyographic, EEG and MRI have been used in human cases and the latter two in sea lions with some degree of discrimination between domoic acid toxicosis and epileptic disease [9].

Neurobehavioral tests have a prominent diagnostic value for humans in identifying selective yet persistent effects on memory [69]. The predominant neurological manifestation from the human amnesic shellfish poisoning event was anterograde memory loss [69,70]. It was observed in 25% of the cases and did not correlate with advanced age. Language function, verbal comprehension and concept formation were within normal range, whereas verbal (Wechsler Memory Scale) and visuospatial (Rey Complex Figure test) memory were both impaired. The impairment was only for delayed recall and not immediate recall, indicating a deficit to retain and/or retrieve new information after it passed out of consciousness. A behavioral diagnostic test in sea lions has been developed based upon habituation of an orienting response to non-aversive auditory stimuli [71]. The test correctly identifies domoic acid toxicity in 50% of subjects, rejects 93% of control subjects, and has a 75% positive predictive value.

The use of genome and proteome profiles has excellent predictive value for rapid diagnostic screening. The use of a canine gene microarray [72] and MALDI-TOF mass spectrometry [73] has been evaluated with serum samples from sea lions in rehabilitation. No individual markers have been shown to have diagnostic value; however, genome and proteome profile assignment by neural networks allowed 100% predictive value.

### 6.3. Comorbidity

#### Cardiomyopathy

A domoic acid-associated degenerative cardiomyopathy has been documented in cases of domoic acid toxicosis in sea lions [24]. Analysis of 102 cases found cardiomyopathy in all age classes of both sexes and in animals with both domoic acid toxicosis and epileptic disease [9]. The primary lesion is a cardiomyocyte vacuolar degeneration and loss with adipocyte replacement [74]. Although a central nervous system origin of disease has been considered, a direct effect on heart via inotropic glutamatergic receptors appears to be the likely origin of domoic acid associated degenerative cardiomyopathy [74,75].

### 6.4. Management and Treatment

#### 6.4.1. Human Poisoning and Disease

In the ASP event, seizures were present in five of the 14 most severe cases which developed focal motor, generalized tonic-clonic and/or partial complex seizures. Seizures in three individuals, including the 84-year-old man who progressed to epileptic disease, were resistant to phenytoin and required high intravenous doses of valium and phenobarbital for control [15]. The 84-year-old case study subject continued phenobarbital pharmacotherapy after his seizures were controlled for a total of six and half months after poisoning. In contrast to his seizures resulting shortly after the poisoning event, the spontaneous complex partial seizures occurring one year after poisoning were responsive to phenytoin, which was used successfully for daily management for the next two years [8].

#### 6.4.2. Sea Lion Poisoning and Disease

Treatment of seizure due to acute domoic acid toxicosis generally requires symptomatic control with benzodiazepines, with lorazepam reported to be more effective than diazepam [2]. Short term management with phenobarbital assists recovery of acute toxicosis cases. By contrast, treatment with the benzodiazepines and phenobarbital was not effective in animals with epileptic disease [9].

#### 6.4.3. Prognosis

The large number of sea lions afflicted by domoic acid poisoning has permitted prognosis following rehabilitation after both acute toxicosis and epileptic disease. Thomas *et al*. [76] examined the outcome of rehabilitated animals released back to the wild equipped with satellite telemetry used to evaluate their movements and dive behavior. Acute animals were three times more likely than control animals to die after release. However, the surviving acute animals did not show any evidence of different navigation or diving behavior compared to controls. By contrast, the chronic animals did not dive as deeply. This was thought to impact their foraging as evidenced by multiple instances of re-stranding in an emaciated state. Chronic animals also traveled greater distances, indicative of deficits in navigational capabilities. This is consistent with previous reports of chronic animals found in atypical locations [9] and may relate to deficits in spatial memory recall reported in humans [69] and working memory in experimental animals [77]. Hence, chronic animals have a substantially poorer prognosis after rehabilitation both in terms of survival behaviors and progression of the epileptic state.

## 7. Conclusions

Domoic acid epileptic disease is characterized by spontaneous recurrent seizures weeks to months after domoic acid poisoning and atypical behaviors in animal subjects, notably conspecific aggression. Precise understanding of the disease state draws on investigations of environmental exposures of humans, sea lions and laboratory exposure of rats. These investigations reveal remarkable corollaries, yet, also, new considerations for the contribution of olfactory and hippocampal pathways to epileptogeneis and maturation of this disease state.

## Supplementary Files

Supplementary File 1 Supplementary Information (PDF, 1650 KB)
