# Supplementary material for: Domoic Acid Epileptic Disease
Source: Mar Drugs. 2014 Mar 6;12(3):1185–207. doi: 10.3390/md12031185 (PMC3967204; doi:10.3390/md12031185)
Supplement: Supplementary File 1 — Supplementary Information (PDF, 1650 KB) [file marinedrugs-12-01185-s001.pdf]

Supplementary Information

Figure S1. Administration regime for DA induced SE. Top photographs show Racine scale stages III through V of rat exposed to domoic acid (Reproduced with permission from [1], © 1990 Society of Toxicologic Pathology). Bottom chart shows the number of seizures of each stage class in late juvenile Sprague Dawley rats treated with an asymptomatic dose (1 mg/kg intraperitoneal) of domoic acid and observed for 1 h. Additional hourly doses were given if no signs of convulsive seizures (modified Racine Class III or higher) were observed. The number of Class III, IV, and V seizures was recorded each hour for a total of three hours after the last dose of domoic acid. Data shown is for an individual exhibiting a typical acute seizure response. Modified from [2].

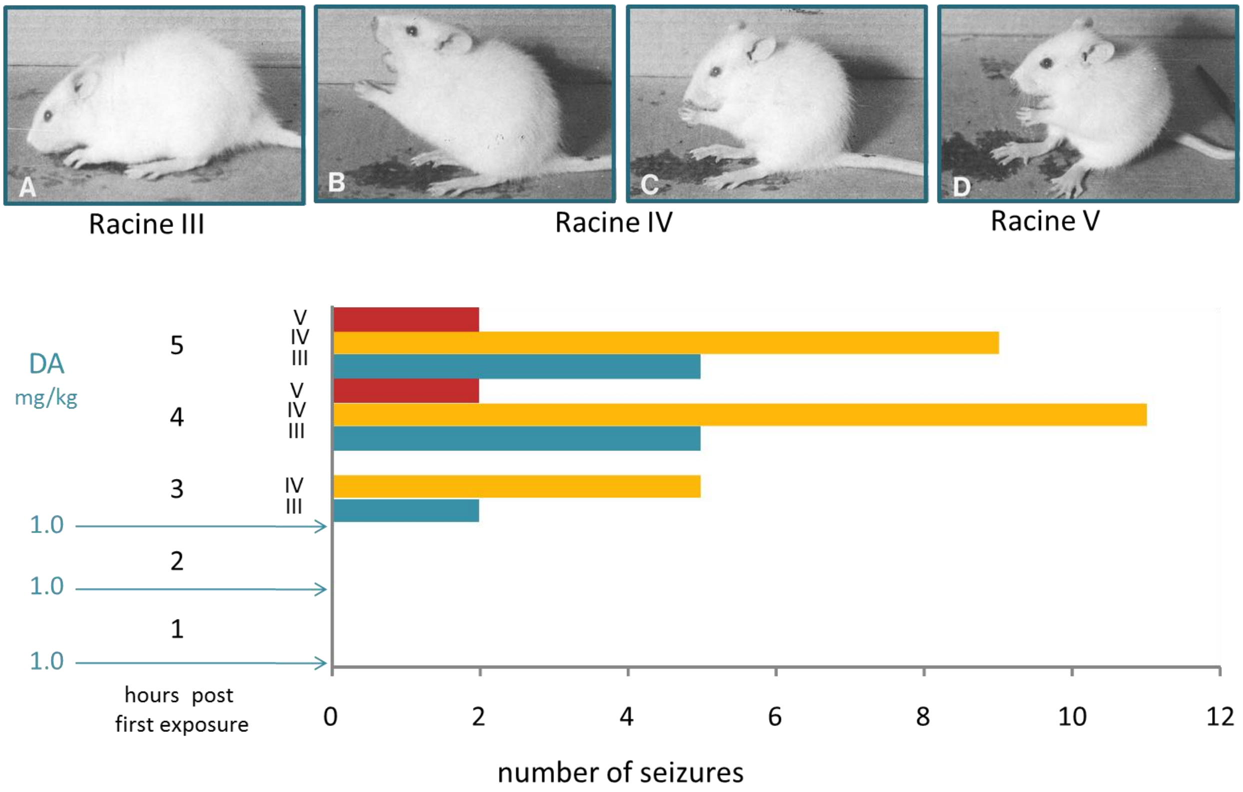

Figure S2. Damage to layers of the olfactory bulb seven days after domoic acid induced status epilepticus. Top left shows approximate level of coronal section taken at bregma 7.3 mm. Top right shows amino cupric silver stained 40 µm section with neutral red counter staining of olfactory bulb and the blue box approximate area of enlargement for bottom panel. Bottom panel shows superficial (left) to deep (bottom) layers: glomerular cell layer, external plexiform layer, mitral cell layer, internal plexiform layer, granule cell layer, and nerve fiber layer; magnification 200×. Modified from [3].

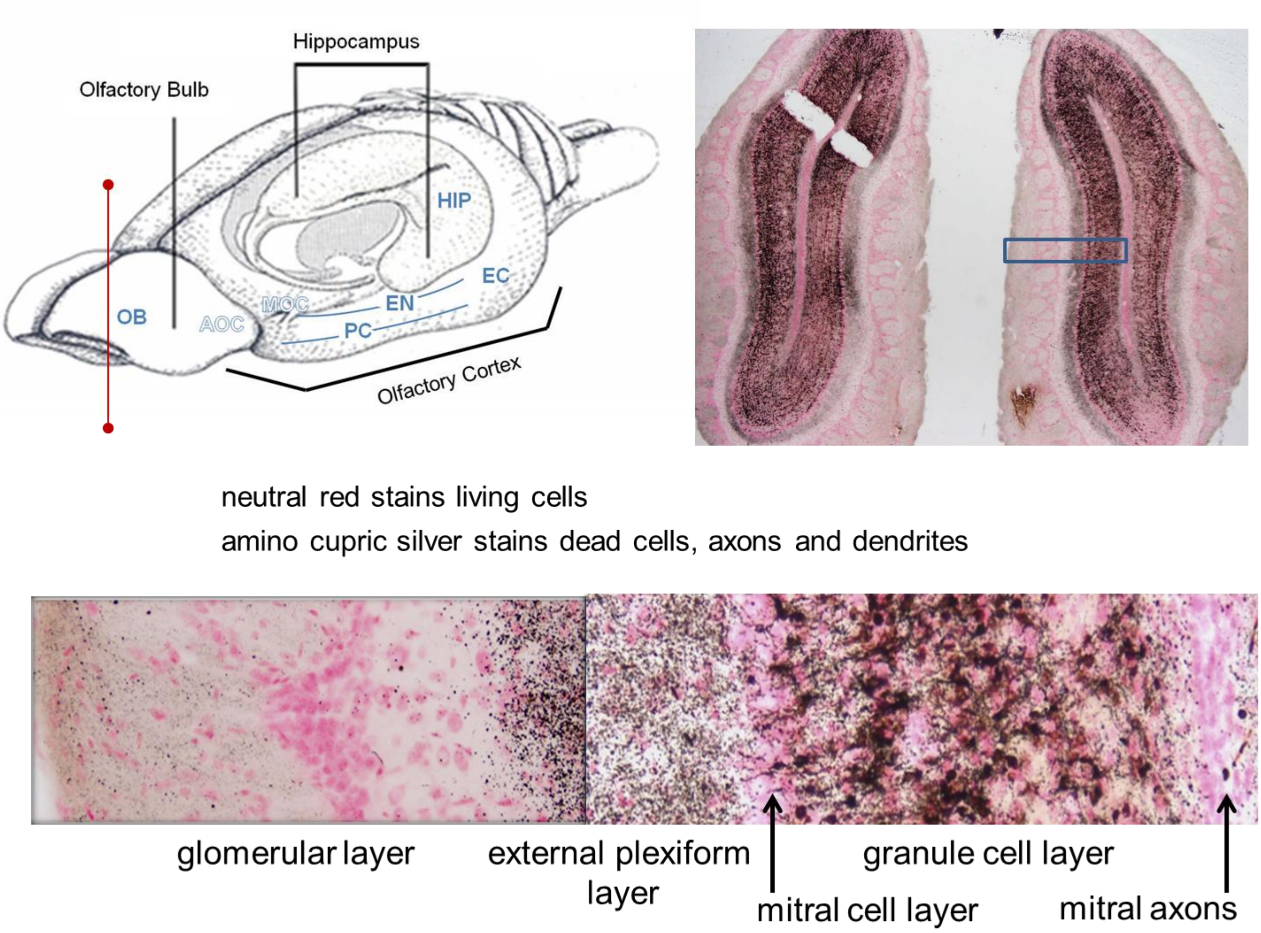

Figure S3. Damage to the anterior olfactory cortex seven days after domoic acid induced status epilepticus. Top left shows approximate level of coronal section taken at bregma 5.2 mm. Bottom panel shows amino cupric silver, red counter staining of degenerated pyramidal cells in the pars principalis and pars externa of the anterior olfactory cortex. Top right shows higher magnification of cupric silver stained pyramidal cells and terminals. Modified from [3].

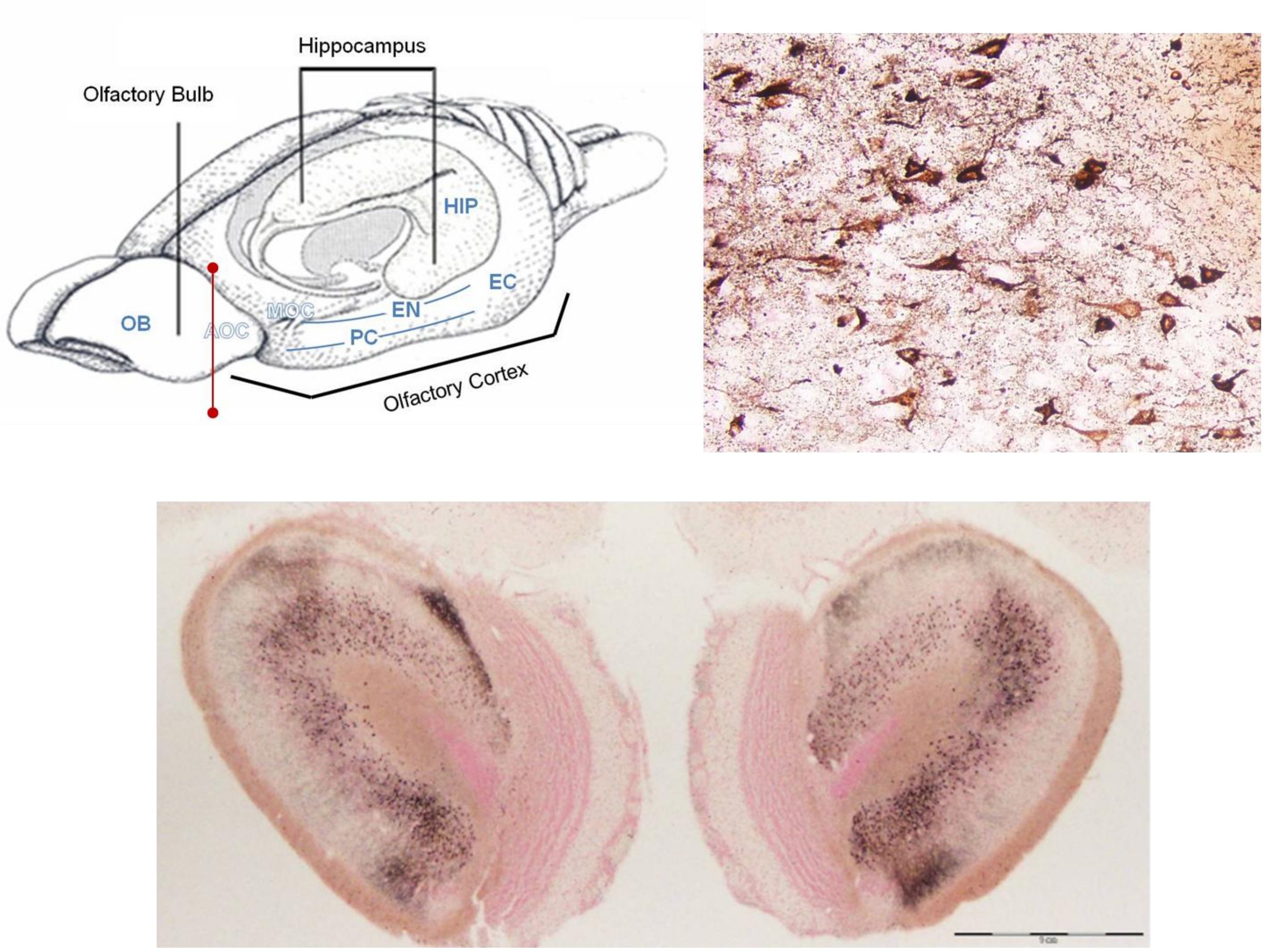

Figure S4. Damage to the medial olfactory cortex seven days after domoic acid induced status epilepticus. Top left shows approximate level of coronal section taken at bregma 3.0 mm. Bottom panel dense stain in the medial olfactory cortex, including the ventral and dorsal tenia (arrows). Stained cells are also evident medial and lateral to the clear capsule of the nucleus accumbens in the anterior olfactory cortex pars posterior and endopirform nucleus, respectively. Some heavier precipitated stain is evident in the left section of the pars posterior. Top right shows higher magnification of cupric silver stained axons with viable neutral red stained cells in background. Modified from [3].

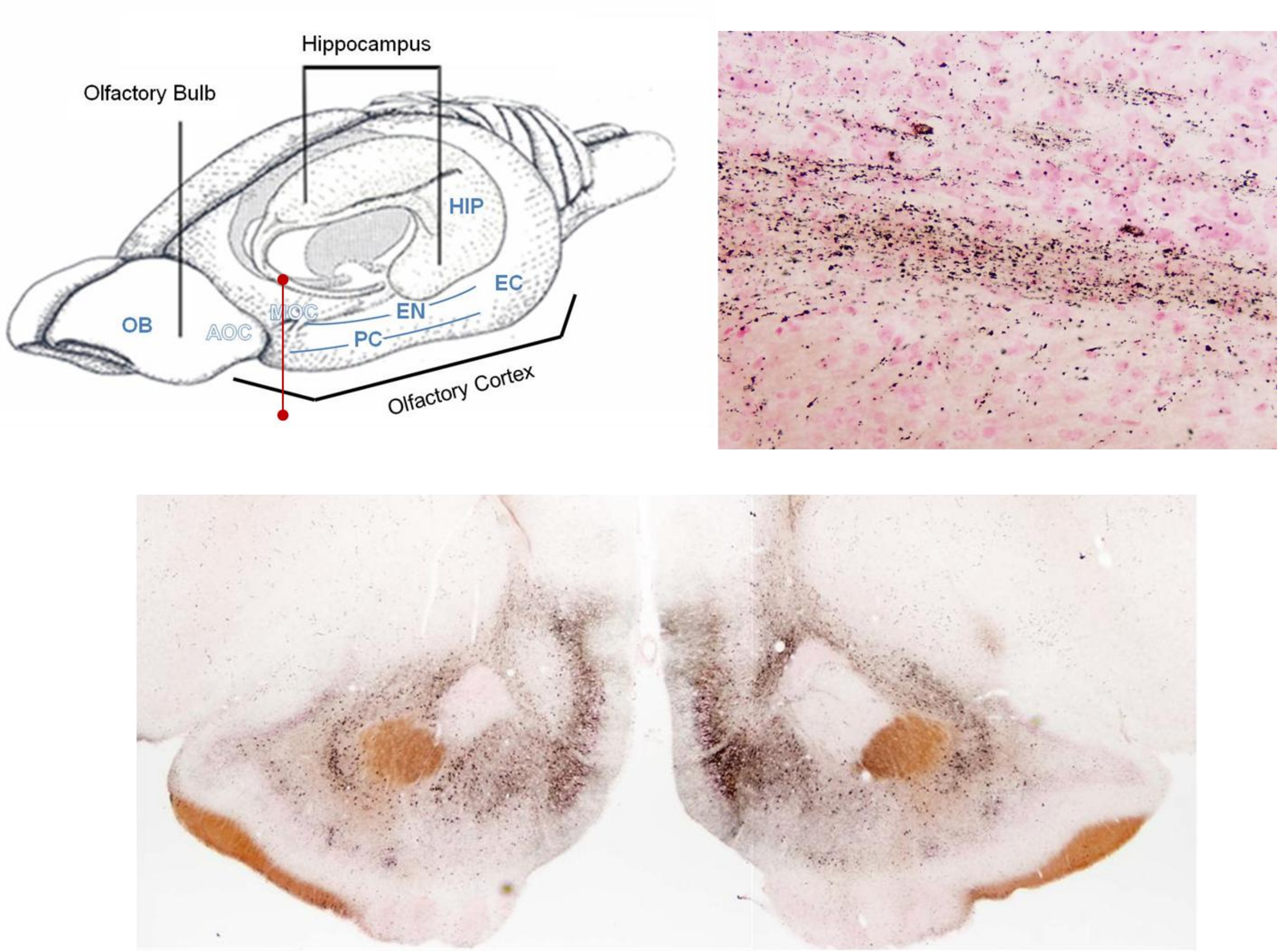

Figure S5. Damage to the olfactory cortex along the lateral olfactory tract seven days after domoic acid induced status epilepticus. Top left shows approximate level of coronal section taken at bregma −0.3 mm. Right panel shows amino cupric silver in the endopiriform nucleus (top arrow) and piriform cortex (bottom arrow). Bottom left shows higher magnification of cupric silver stained pyramidal cells and terminals in piriform cortex. Modified from [3].

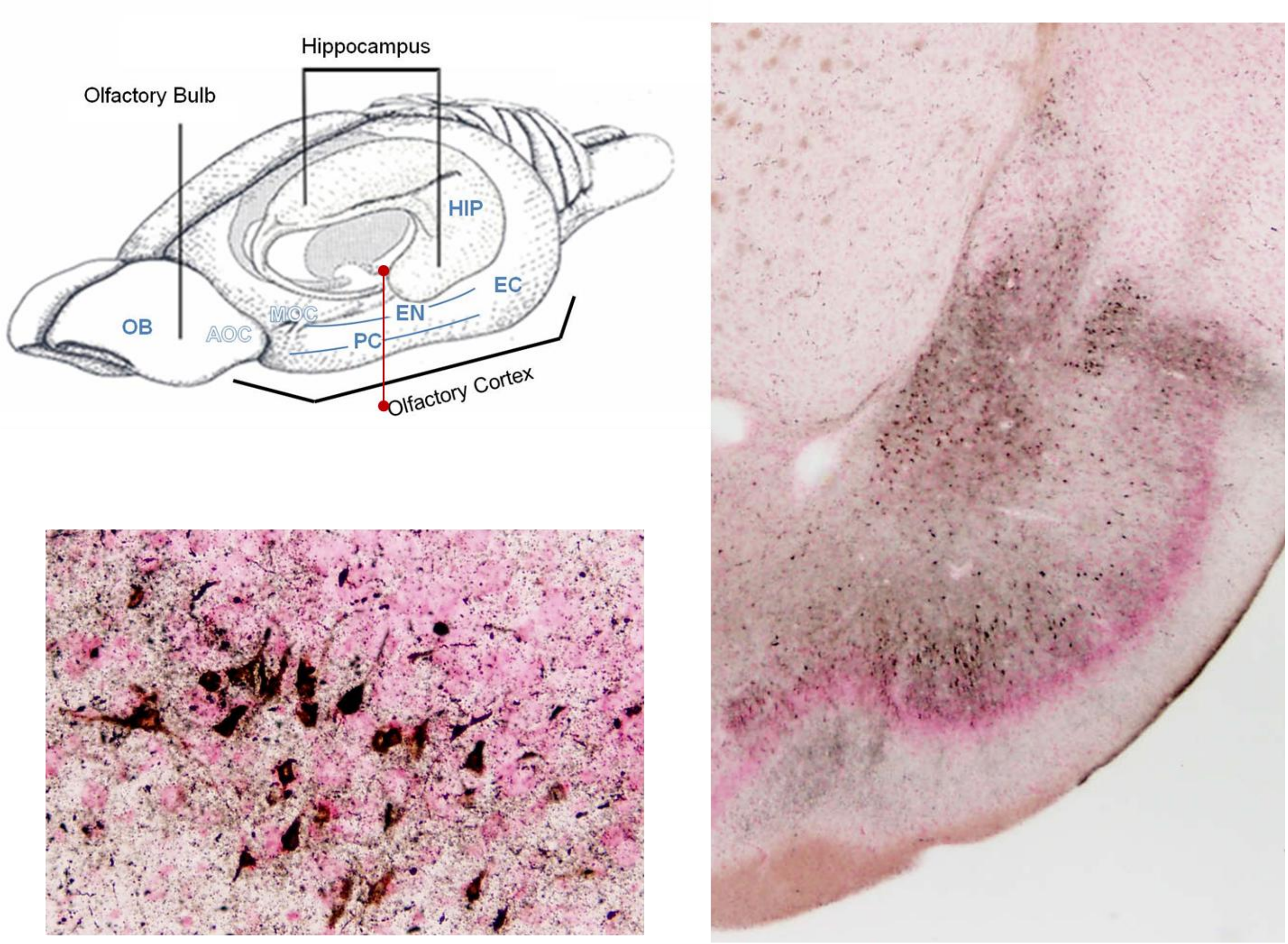

Figure S6. Damage to the posterior extent of the entorhinal cortex seven days after domoic acid induced status epilepticus. Top left shows approximate level of coronal section taken at bregma −8.0 mm. Right panel shows amino cupric silver stain in distinct layers with heavy cell body and terminal staining evident in layer III. Bottom left shows higher magnification of cupric silver stained pyramidal cells and terminals in layer III of entorhinal cortex. Modified from [3].

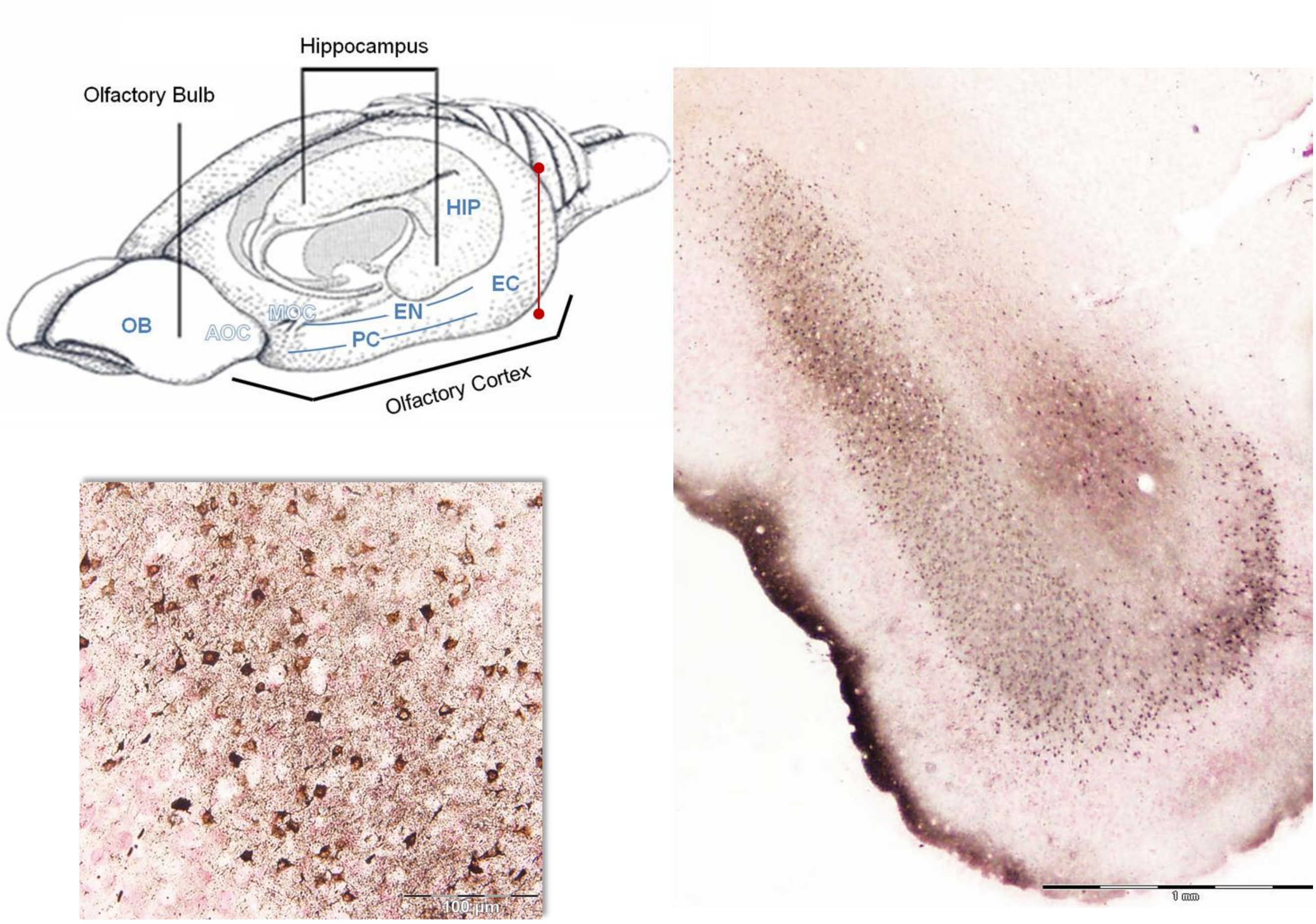

Figure S7. Damage to the posterior region of the olfactory cortex along the lateral olfactory tract in epileptic animal twelve weeks after domoic acid induced status epilepticus. Top left shows approximate level of coronal section taken at bregma −0.3 mm. Right panel shows amino cupric silver in cells of the piriform cortex. Bottom left shows higher magnification of cupric silver stained pyramidal cells and terminals in piriform cortex. Modified from [4].

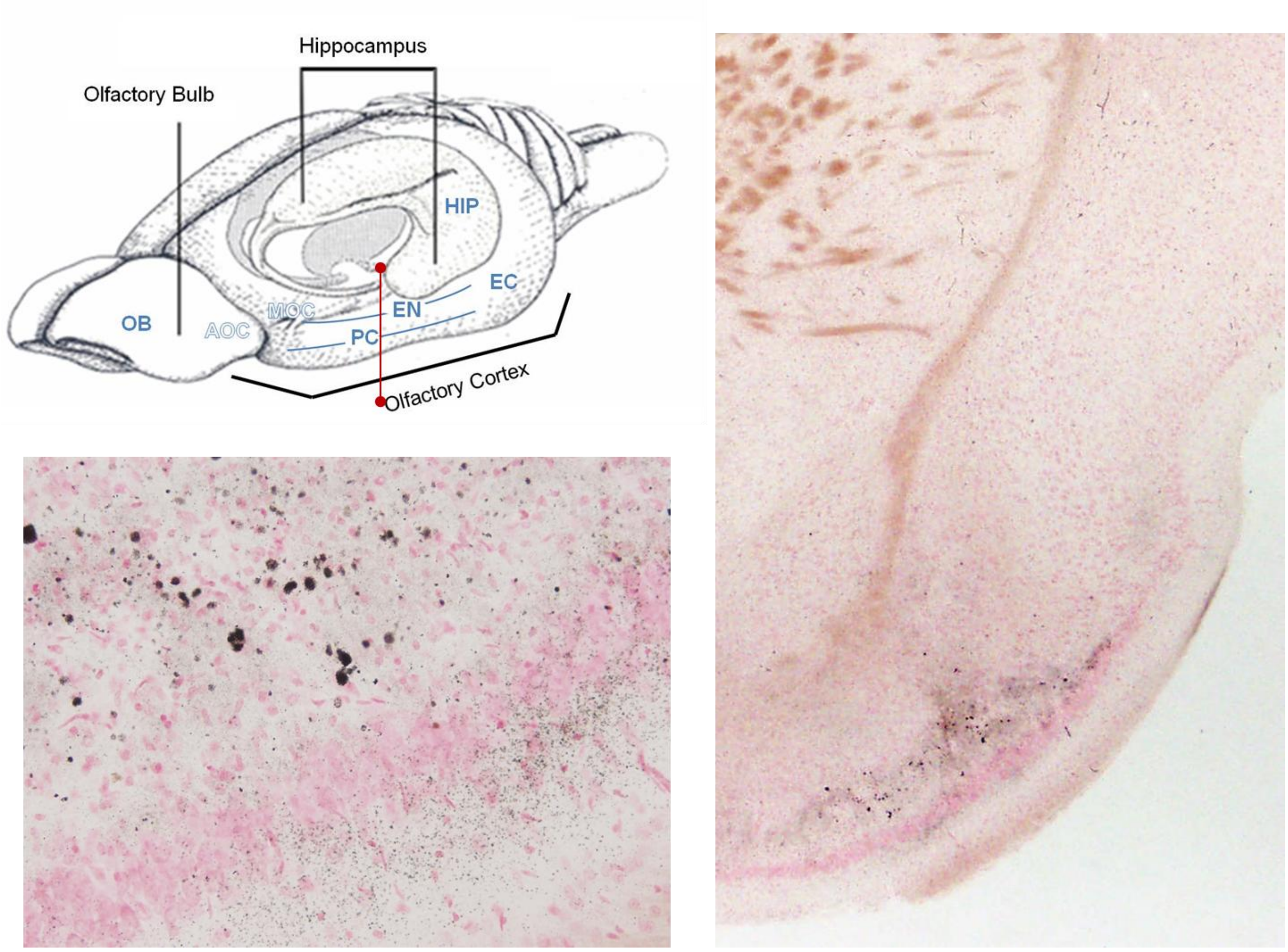

Figure S8. Damage to the posterior region of the olfactory cortex along the lateral olfactory tract twelve weeks after domoic acid induced status epilepticus. Top left shows approximate level of coronal section taken at bregma −0.3 mm. Right panel shows Nissl stained cells of the piriform cortex in control (top) and depleted deep layers of cells in seizing (middle) and atypical aggressive (bottom) animals. Bottom left shows cell counts from layer 3 of the piriform cortex in matched sections at roughly Bregma −0.4 averaged from both left and right hemispheres. Control (112) depicted as a diamond. Modified from [4].

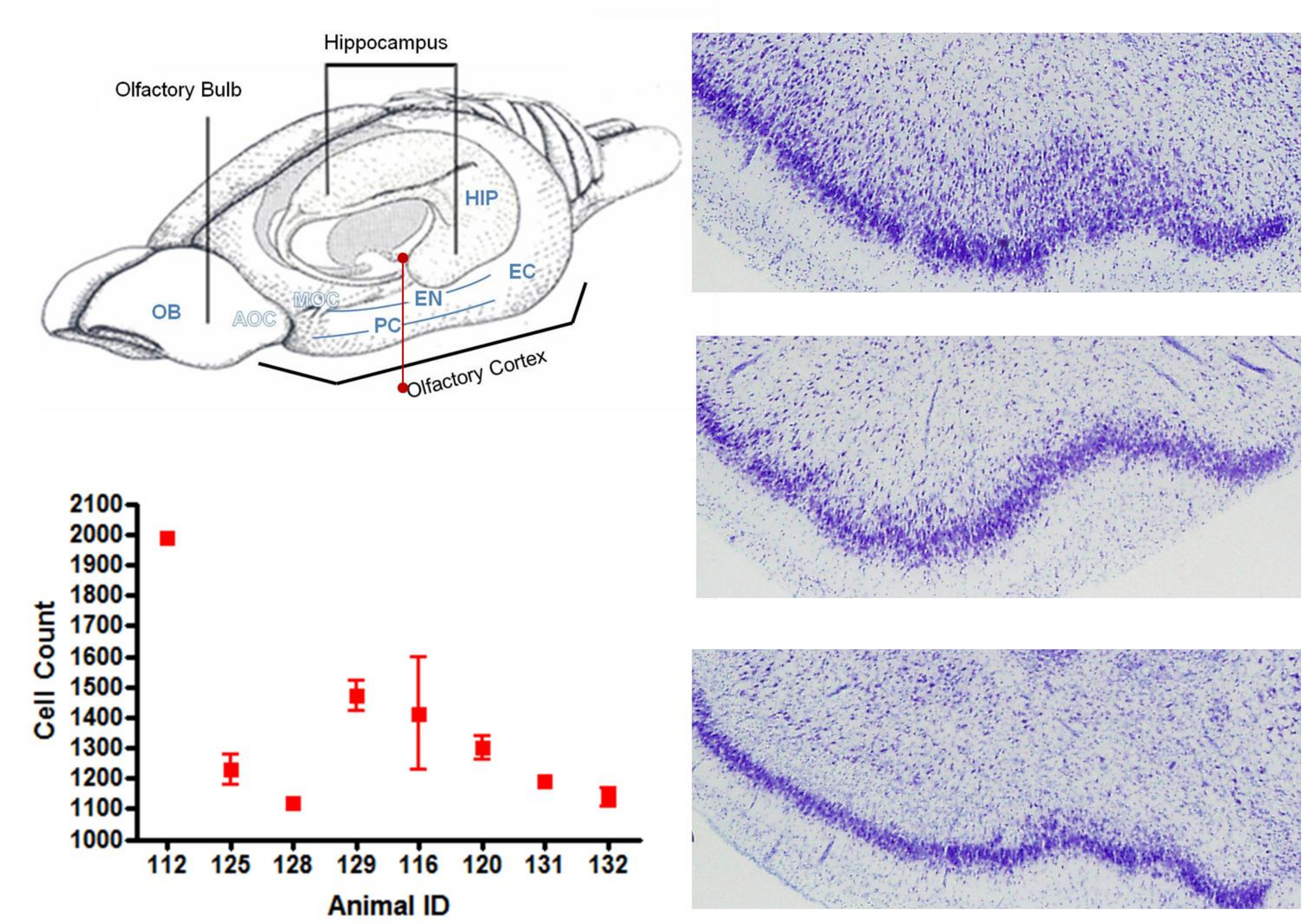

References

1. Tryphonas, L.; Iverson, F. Neuropathology of excitatory neurotoxins: the domoic acid model. *Toxicol. Pathol.* **1990**, *18*, 165–169.
2. Muha, N.; Ramsdell, J.S. Domoic acid induced seizures progress to a chronic state of epilepsy in rats. *Toxicon* **2011**, *57*, 168–171.
3. Tiedeken, J.A.; Muha, N.; Ramsdell, J.S. A cupric silver histochemical analysis of domoic acid damage to olfactory pathways following status epilepticus in a rat model for chronic recurrent spontaneous seizures and aggressive behavior. *Toxicol. Pathol.* **2012**, doi:10.1177/0192623312453521.
4. Tiedeken, J.A.; Ramsdell, J.S. Persistent neurological damage associated with spontaneous recurrent seizures and atypical aggressive behavior of domoic acid epileptic disease. *Toxicol. Sci.* **2013**, *133*, 133–143.

© 2014 by the authors; licensee MDPI, Basel, Switzerland. This article is an open access article distributed under the terms and conditions of the Creative Commons Attribution license (http://creativecommons.org/licenses/by/3.0/).
